# Deep Learning and Its Applications in Biomedicine

**DOI:** 10.1016/j.gpb.2017.07.003

**Published:** 2018-03-06

**Authors:** Chensi Cao, Feng Liu, Hai Tan, Deshou Song, Wenjie Shu, Weizhong Li, Yiming Zhou, Xiaochen Bo, Zhi Xie

**Affiliations:** 1 CapitalBio Corporation, Beijing 102206, China; 2 Department of Biotechnology, Beijing Institute of Radiation Medicine, Beijing 100850, China; 3 State Key Lab of Ophthalmology, Zhongshan Ophthalmic Center, Sun Yat-sen University, Guangzhou 500040, China; 4 Zhongshan School of Medicine, Sun Yat-sen University, Guangzhou 500040, China; 5 Department of Biomedical Engineering, Medical Systems Biology Research Center, Tsinghua University School of Medicine, Beijing 100084, China

**Keywords:** Deep learning, Big data, Bioinformatics, Biomedical informatics, Medical image, High-throughput sequencing

## Abstract

Advances in biological and medical technologies have been providing us explosive volumes of biological and physiological data, such as **medical images**, electroencephalography, genomic and protein sequences. Learning from these data facilitates the understanding of human health and disease. Developed from artificial neural networks, **deep learning**-based algorithms show great promise in extracting features and learning patterns from complex data. The aim of this paper is to provide an overview of deep learning techniques and some of the state-of-the-art applications in the biomedical field. We first introduce the development of artificial neural network and deep learning. We then describe two main components of deep learning, *i.e.*, deep learning architectures and model optimization. Subsequently, some examples are demonstrated for deep learning applications, including medical image classification, genomic sequence analysis, as well as protein structure classification and prediction. Finally, we offer our perspectives for the future directions in the field of deep learning.

## Introduction

Deep learning is a recent and fast-growing field of machine learning. It attempts to model abstraction from large-scale data by employing multi-layered deep neural networks (DNNs), thus making sense of data such as images, sounds, and texts [1]. Deep learning in general has two properties: (1) multiple layers of nonlinear processing units, and (2) supervised or unsupervised learning of feature presentations on each layer [1]. The early framework for deep learning was built on artificial neural networks (ANNs) in the 1980s [2], while the real impact of deep learning became apparent in 2006 [3], [4]. Since then, deep learning has been applied to a wide range of fields, including automatic speech recognition, image recognition, natural language processing, drug discovery, and bioinformatics [5], [6], [7].

The past decades have witnessed a massive growth in biomedical data, such as genomic sequences, protein structures, and medical images, due to the advances of high-throughput technologies. This deluge of biomedical big data necessitates effective and efficient computational tools to store, analyze, and interpret such data [5], [8]. Deep learning-based algorithmic frameworks shed light on these challenging problems. The aim of this paper is to provide the bioinformatics and biomedical informatics community an overview of deep learning techniques and some of the state-of-the-art applications of deep learning in the biomedical field. We hope this paper will provide readers an overview of deep learning, and how it can be used for analyzing biomedical data.

### The development of ANNs

As a basis for deep learning, ANNs were inspired by biological processes in the 1960s, when it was discovered that different visual cortex cells were activated when cats visualized different objects [9], [10]. These studies illustrated that there were connections between the eyes and the cells of the visual cortex, and that the information was processed layer by layer in the visual system. ANNs mimicked the perception of objects by connecting artificial neurons within layers that could extract the features of objects [11], [12], [13], [14], [15], [16]. However, ANN research stagnated after the 1960s, due to the low capability resulting from its shallow structures and the limited computational capacity of computers at that time [17].

**Figure 1:**
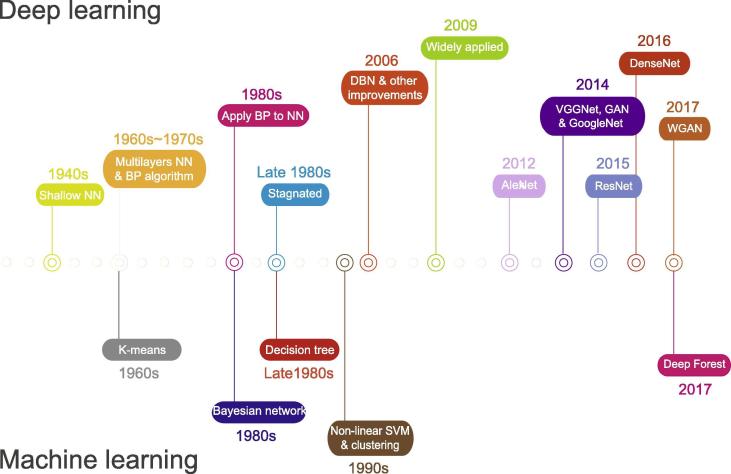
**Timeline of the development of deep learning and commonly-used machine learning algorithms**The development of deep learning and neural networks is shown in the top panel, and several commonly-used machine learning algorithms are shown in the bottom panel. NN, neural network; BP, backpropagation; DBN, deep belief network; SVM, support vector machine; AE: auto-encoder; VAE: variational AE; GAN: generative adversarial network; WGAN: Wasserstein GAN.

Thanks to the improvement in computer capabilities and methodologies [18], ANNs with efficient backpropagation (BP) facilitated studies on pattern recognition [19], [20], [21], [22], [23]. In a neural network with BP, classifications were first processed by the ANN model, and weights were then modified by evaluating the difference between the predicted and the true class labels. Although BP helped to minimize errors through gradient descent, it seemed to work only for certain types of ANNs [24]. Through improving the steeper gradients with BP, several learning methods were proposed, such as momentum [25], adaptive learning rate [26], [27], [28], least-squares methods [29], [30], quasi-Newton methods [31], [32], [33], [34], and conjugate gradient (CG) [35], [36]. However, due to the complexity of ANNs, other simple machine learning algorithms, such as support vector machines (SVMs) [37], random forest [38], [39], and k-nearest neighbors algorithms (k-NN) [40], gradually overtook ANNs in popularity (Figure 1).

### The development of deep learning

An ANN with more hidden layers offers much higher capacity for feature extraction [4]. However, an ANN often converges to the local optimum, or encounters gradient diffusion when it contains deep and complex structures [41]. A gradient propagated backwards rapidly diminishes in magnitude along the layers, resulting in slight modification to the weights in the layers near the input (http://deeplearning.stanford.edu/wiki/index.php/UFLDL_Tutorial) [42]. Subsequently, a layer-wise pre-training deep auto-encoder (AE) network was proposed, bringing ANNs to a new stage of development [3], [4], [43], [44], [45] (Figure 1). In this network, each layer is trained by minimizing the discrepancy between the original and the reconstructed data [4]. The layer-wise pre-training breaks the barrier of gradient diffusion [4], and also results in a better choice of weights for deep neural networks (DNNs), thereby preventing the reconstructed data from reaching a local optimum where the local optimum is usually caused by the random selection of initial weights. In addition, the employment of graphic processing units (GPUs) also renews the interest of researchers in deep learning [46], [47].

With the focus of more attention and efforts, deep learning has burgeoned in recent years and has been applied broadly in industry. For instance, deep belief networks (DBNs) and stacks of restricted Boltzmann machines (RBMs) [3], [48], [49] have been applied in speech and image recognition [3], [45], [50] and natural language processing [51]. Proposed to better mimick animals’ perceptions of objects [52], convolutional neural networks (CNN) have been widely applied in image recognition [53], [54], [55], image segmentation [56], video recognition [57], [58], and natural language processing [59]. Recurrent neural networks (RNNs) are another class of ANNs that exhibit dynamic behavior, with artificial neurons that are associated with time steps [25], [60], [61]. RNNs have become the primary tool for handling sequential data [62], and have been applied in natural language processing [63] and handwriting recognition [64]. Later on, variants of AEs, including sparse AEs, stacked AEs (SAEs), and de-noising AEs, have also gained popularity in pre-training deep networks [49], [65], [66], [67].

Although applications of deep learning have been primarily focused on image recognition, video and sound analyses, as well as natural language processing, it also opens doors in life sciences, which will be discussed in detail in the next sections.

## Brief description of deep learning

Although the underlying assumptions and theories are different, the basic idea and processes for feature extraction in most deep NN (DNN) architectures are similar. In the forward pass, the network is activated by an input to the first layer, which then spreads the activation to the final layer along the weighted connections, and generates the prediction or reconstruction results. In the backward pass, the weights of connections are tuned by minimizing the difference between the predicted and the real data.

### Basic concepts

#### Activation functions

Activation functions form the non-linear layers in all deep learning frameworks; and their combinations with other layers are used to simulate the non-linear transformation from the input to the output [62]. Therefore, better feature extraction can be achieved by selecting appropriate activation functions [7], [68], [69]. Here, we introduce several commonly-used activation functions, represented by *g*.

• Sigmoid function: $g(a)=\frac{1}{1+e^{-a}}$, where *a* is the input from the front layer. A sigmoid function transforms variables to values ranging from 0 to 1 and is commonly used to produce a Bernoulli distribution. For example:

$$\overset{\sim }{g}=\left\{\begin{array}{cc} 0 & \text{if}\;g(a)⩽0.5\\ 1 & \text{if}\;g(a)>0.5 \end{array}\right)\text{,}$$

• Hyperbolic tangent: $g(a)=\text{tan h}(a)=\frac{e^{a}-e^{-a}}{e^{a}+e^{-a}}$. Here, the derivative of *g* is calculated as $g^{\prime }=1-g^{2}$, making it easy to work with in BP algorithms.
• Softmax: $g(a)=\frac{e^{a_{i}}}{\sum_{j}e^{a_{j}}}$. The softmax output, which a_n_ be considered as a probability distribution over the categories, is commonly used in the final layer.
• Rectified linear unit (ReLU): g(a)=max(0,a). This activation function and its variants show superior performance in many cases and are the most popular activation function in deep learning so far [68], [70], [71], [72]. ReLU can also solve the gradient diffusion problem [73], [74].
• Softplus: $g(a)=\text{log}(1+e^{a})$. This is one of the variants of ReLU, representing a smooth approximation of ReLU (in this article, the log always represents the natural logarithm).
• Absolute value rectification: g(a)=|a|. This function is useful when the pooling layer takes the average value in CNNs [75], thus preventing otherwise the negative features and the positive features from diminishing.
• Maxout: $g_{i}(x)=\underset{i}{\max}(b_{i}+w_{i}\cdot x)$. The weight matrix in this function is a three-dimensional array, where the third array corresponds to the connection of the neighboring layers [76].

#### Optimization objective

An optimization objective is often composed of a loss function and a regularization term. The loss function measures the discrepancy between the output of the network depend on model parameters (θ) f(x|θ) and the expected result y, e.g., the true class labels in classification tasks, or the true level in prediction tasks. However, a good learning algorithm performs well not only on the training data, but also on the test data. A collection of strategies designed to reduce the test error is called regularization [62]. Some regularization terms apply penalties to parameters to prevent overly complex models. Here, we briefly introduce the commonly used loss function L(f(x|θ),y) and regularization term Ω(θ). The optimization objective is usually defined as:

(1) $$\overset{\sim }{L}(X\text{,}y\text{,}\theta )=L(f(x|\theta )\text{,}y)+\alpha \Omega (\theta )$$

where α is a balance of these two components, and in practice, the loss function is usually calculated across randomly-sampled training samples rather than the data-generating distribution, since the latter is unknown.

##### Loss function

Most DNNs use cross entropy between the training data and the model distribution as the loss function. The most commonly used form of cross entropy is the negative conditional log-likelihood: L(f(x|θ),y)=-logP(f=y|x,θ). This is a collection of loss functions corresponding to the distribution of *y* given the value of input variable *x*. Here, we introduce several commonly used loss functions that follow this pattern:

Suppose *y* is continuous and has a Gaussian distribution over a given variable *x*. The loss function would be:

(2) $$L(f(x|\theta )\text{,}y)=-\log\left[\sqrt{\frac{1}{2\pi \sigma^{2}}}\exp\left(-\frac{1}{2\sigma^{2}}{\left(y-f\right)}^{2}\right)\right]=\frac{1}{2\sigma^{2}}{\left(y-f\right)}^{2}+\frac{1}{2}\log(2\pi \sigma^{2})$$

Which is equivalently described as the squared error. The squared error was the most commonly used loss function in the 1980s [62]. However, it often tends to penalize outliers excessively, leading to slower convergence rates [77].

If *y* follows the Bernoulli distribution, then the loss function will be:

(3) L(f(x|θ),y)=-ylogf(x|θ)-(1-y)log(1-f(x|θ))

When *y* is discrete and has only two values, for instance, y∈{1,2,…,k}, we can take the softmax value (see commonly-used activation functions) as the probability over the categories. Then the loss function will be:

(4) $$L(f(x|\theta )\text{,}y)=-\log\left(\frac{e^{a_{y}}}{\sum_{j}e^{a_{j}}}\right)=-a_{y}+\log\left(\underset{j}{\sum }e^{a_{j}}\right)$$

##### Regularization term

$L^{2}$ parameter regularization is the most common form of regularization term and contributes to the convexity of the optimization objective, leading to an easy solution for the minimum using the Hessian matrix [78], [79]. $L^{2}$ parameter regularization can be defined as

(5) $$\Omega (\theta )=\frac{1}{2}\|\omega {\|}^{2}$$

where Ω represents weights of connecting units in the network (the same as in the following context).

Compared to $L^{2}$ parameter regularization, $L^{1}$ parameter regularization results in a sparser solution of ω and tends to learn small groups of features. $L^{1}$ parameter regularization can be defined as

(6) $$\Omega (\theta )=\|\omega {\|}_{1}=\underset{i}{\sum }|\omega_{i}|$$

Frobenius parameter regularization is induced by the inner product and is block decomposable, therefore it is easier to compute [80], [81]. Frobenius parameter regularization can be defined as

(7) $$\omega (\theta )=\sqrt{\underset{i}{\sum }\underset{j}{\sum }|\omega_{\mathrm{ij}}{|}^{2}}=\sqrt{\overset{\mathrm{rank}(\omega )}{\underset{i=1}{\sum }}\sigma_{i}^{2}}$$

where $\sigma_{i}$ is the *i*-th largest singular value. Frobenius parameter regularization has a function similar to nuclear norm in terms of regularization.

Nuclear norm has been widely used as regularization in recent years [82], [83], [84]. Nuclear norm regularization measures the sum of the singular values of ω and can be defined as

(8) $$\Omega (\theta )=\|\omega {\|}_{*}=\overset{\mathrm{rank}(\omega )}{\underset{i=1}{\sum }}\sigma_{i}$$

#### Optimization methods

A learning task is transformed to an optimization problem, to achieve the minima of the objective function by selecting appropriate hyperparameters. The basic processes of different optimization methods are similar. First, the output $f=f(x|\theta_{0})$ and the optimization objective $\overset{\sim }{L}$ of the model are computed using the initial parameters $\theta_{0}$. The network parameters θ are then tuned to decrease the objective function value from the final layer to the first layer [18]. This process is repeated until the proper model and a small fit error, *i.e.*, loss function value, are obtained (http://deeplearning.stanford.edu/wiki/index.php/UFLDL_Tutorial).

However, different optimization methods have different advantages and disadvantages on different architectures and loss functions [62], [85]. Stochastic gradient descent (SGD) and its variants are the most-used methods, which update the parameters by a gap corresponding to the Jacobian matrix. The computation time per update does not grow too much even with a large training set [86], [87], [88]. AdaGrad updates parameters according to the accumulation of squared gradients, which can converge rapidly when applied to convex functions, but performs worse in certain models [62]. RMSProp, an AdaGrad algorithm, has been an effective and popular method for parameter optimization. Another type of algorithm makes use of second order derivatives to improve optimization. For instance, limited-memory Broyden–Fletcher–Goldfarb–Shanno algorithm (BFGS) is one type of quasi-Newton method, which iteratively refines the approximation of the inverse of the Hessian matrix and avoids storing the matrix. BFGS is good at dealing with low dimensionality problems, particularly for convolutional models [85]. In addition, conjugate gradient combines conjugacy and gradient descent in the update direction decision for parameters, efficiently avoiding the calculation of the inverse Hessian [4], [35], [36], while contrastive divergence is usually used in RBM model [89], [90], [91]. With the help of a GPU [47], many algorithms can be accelerated significantly [85].

The proper architecture and objective function should be selected according to data considered. As a type of machine learning, deep learning can also encounter “overfitting,” that is, low error on training data but high error on test data. In addition to the regularization terms, other methods for regularization are also important for reducing test error. Adding noise to the input or to the weights are efficient regularization strategies [41], [92], as in the case of a denoising AE [93]. Stopping the optimization early by setting an iteration number is another commonly used strategy to prevent the network from overfitting [62]. Parameter sharing, just like in CNN, can also contribute to regularization [94]. Dropout can force units to independently evolve, and randomly remove portions of units in ANN on each iteration, and can therefore achieve better results with inexpensive computation [73], [95], [96].

### Deep learning architectures

#### AEs

Different from ordinary ANNs, AEs extract features from unlabeled data and set target values to be equal to the inputs [4], [49], [97]. Given the input vector $\{x^{\left(1\right)}\text{,}x^{\left(2\right)}\text{,}x^{\left(3\right)}\text{,}\ldots \}\text{,}x^{\left(i\right)}\in R^{n}$, the AE tries to learn the model:

(9) $$h_{w\text{,}b}(x)=g(\mathrm{Wx}+b)\approx x$$

where *W* and *b* are the parameters of the model, *g* is the activation function (same definition applied in the following context), and $h_{W\text{,}b}$ represents the hidden units. When the number of hidden units, which represents the dimension of features, is smaller than the input dimension, the AE performs a reduction of data dimensionality similar to principal component analysis [98]. Besides pattern recognition, an AE with a classifier in the final layer can perform classification tasks as well.

#### RBMs and DBNs

RBMs are generative graphical models that aim to learn the distribution of training data. Since we do not know which distribution the data obeys, we cannot directly compute model parameters using the maximum likelihood principle. Boltzmann machines (BMs) use an energy function to generate the probability distribution (see Equations (12), (13) below), and then optimize parameters until the model learns the true distribution of the data. The original BMs have not been demonstrated to be useful for practical problems, while RBMs are commonly used in deep learning.

RBMs restrict the BMs to a bipartite graph, *i.e.*, there are no connections within visible units ν=x or hidden units ℏ. This restriction ensures the conditional independency of hidden units and visible units [91], *i.e.*,

(10) $$\begin{array}{cc} & P(h|v)=\Pi_{i}p(h_{i}|v)\\ & P(v|h)=\Pi_{j}p(v_{j}|h) \end{array}$$

Furthermore, most RBMs rely on the assumption that all units in the network take only one of the two possible values 0 or 1, *i.e.*, $\nu_{j}\text{,}h_{i}\in (0\text{,}1)$. Provided with the activation function, the conditional distribution of hidden and visible units can be expressed in the following form:

(11) $$\begin{array}{cc} & p(h_{i}=1|v)=g(W_{i}v+c_{i})\\ & p(v_{j}=1|h)=g(W_{j}^{\prime }h+b_{j}) \end{array}$$

According to the Boltzmann distribution, probability distributions over hidden and visible vectors are defined as:

(12) $$P(\nu \text{,}h)=\frac{1}{Z}e^{-E(\nu \text{,}h)}$$

where $Z=\sum e^{-E(\nu \text{,}h)}$ is the normalizing constant and $E(v\text{,}h)=-b^{\prime }v-c^{\prime }h-h^{\prime }\mathrm{Wv}$ is the energy function [99]. The conditional probability distribution can also be computed by integral, and the parameters can then be optimized by minimizing the Kullback-Leibler divergence.

Overall, given the network architectures and optimized parameters, the distribution of the visible units could be computed as:

(13) $$P(v)=\underset{h}{\sum }p(v\text{,}h)=\underset{h}{\sum }\frac{e^{-E(v\text{,}h)}}{Z}$$

A DBN can be viewed as a stack of RBMs [6], [24], [100] or AEs [66], [101]. Similar to RBMs, DBNs can learn the distribution of the samples, or learn to classify the inputs given class labels [3]. However, the p(h) in the formula $p(v)=\sum_{h}p(v\text{,}h)=\sum_{h}p(h)p(v/h)$ is replaced by a better model after the weight of connections *W* is learned by an RBM [3], [100].

In addition to feature extraction, RBMs can also learn distributions of unlabeled data as generative models, and classify labeled data as discriminative models (regard the hidden units as labels). Similar to AEs, RBMs can also pre-train parameters for a complex network.

#### Convolutional neural networks

Different from other deep learning structures, artificial neurons in convolutional neural networks (CNNs) extract features of small portions of input images, which are called receptive fields. This type of feature extraction was inspired by the visual mechanisms in living organisms, where cells in the visual cortex are sensitive to small regions of the visual field [52], [102].

**Figure 2:**
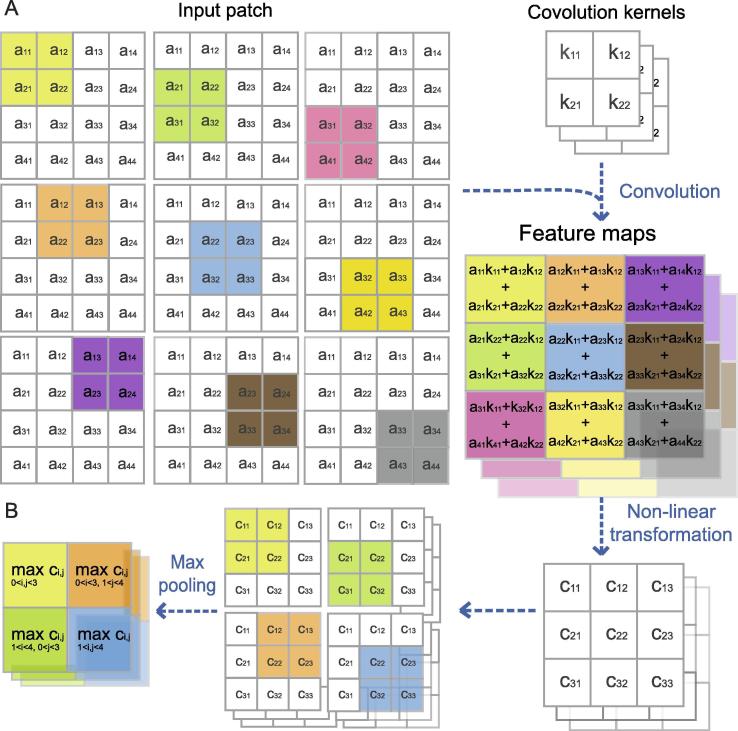
**Illustration of convolutional neural network** **A.** In the convolution layer, fields (different color blocks in the table) of the input patch (represented by a) are multiplied by matrices (convolution kernel, represented by k). **B.** In the pooling layer, the results of convolution are summarized (the max pooling is taken as example here). a_ij_, c_ij_, k_ij_ represent the number located in line i and column j in the corresponding matrix.

Besides the activation function, there are two particular types of layers in CNNs: the convolutional layer and the pooling layer (Figure 2). In the convolutional layer, the image is convolved by different convolutional filters via shifting the receptive fields step by step [87] (Figure 2A). The convolutional filters share the same parameters in every small portion of the image, largely reducing the number of hyperparameters in the model. A pooling layer, taking advantage of the “stationarity” property of images, takes the mean, the max, or other statistics of the features at various locations in the feature maps, thus reducing the variance and capturing essential features (http://deeplearning.net/tutorial/lenet.html) (Figure 2B).

#### Recurrent neural networks

Recurrent neural networks (RNNs) outperform other deep learning approaches in dealing with the sequential data. Based on the property of sequential data, parameters across different time steps of the RNN model are shared. Taking speech as an example: some vowels may last longer than other sounds; the difference makes absolute time steps meaningless and demands that the model parameters be the same among the time steps [62].

**Figure 3:**
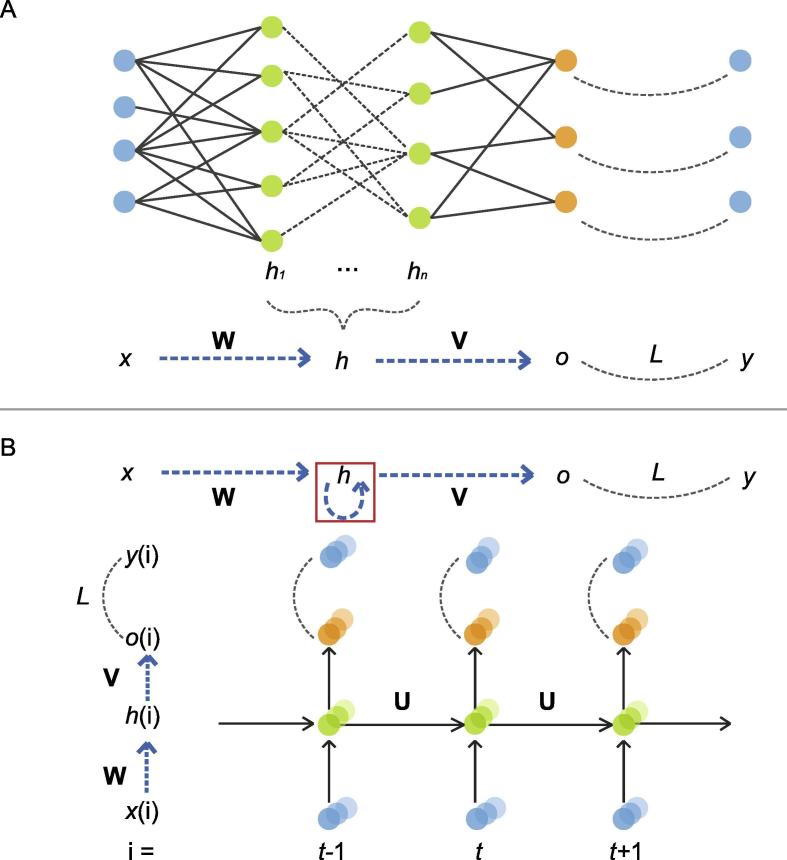
**Illustration of recurrent neural network** **A.** The unfold form of common neural networks (top) and schema (bottom). **B.** An illustration of recurrent neural networks (top) and their unfold form (bottom). The red square represents one time step delay. Different from panel A, the arrows in panel B represent sets of connections. W and B represent the weight matrix and bias vector, respectively. x and y represent the input and output of the network, respectively; h indicates the hidden units of network; L consists of couples of transformations, such as densely-connected layers or dropout layers; U indicates the transformation between two neighbor time points; and t represents the time point.

Beside the parameter sharing, RNNs are different from other multilayer networks by virtue of having a circuit, which represents hidden-to-hidden recurrence. A simple recurrent network corresponds to the following equation:

(14) $$\begin{array}{cc} & h_{\left(t\right)}=g(b+{\mathrm{Uh}}_{\left(t-1\right)}+{\mathrm{Wx}}_{\left(t\right)})\\ & o_{\left(t\right)}=c+{\mathrm{Vh}}_{\left(t\right)} \end{array}$$

where *t* is the label for time, *W* and *V* represent the weights connecting hidden and input units, and hidden and output units, respectively, *b* and *c* are the offsets of the visible and hidden layers, respectively, *g* is the activation function, and *U* represents the weights connecting hidden units at time t-1 to hidden units at time *t* (Figure 3).

Similar to other deep learning architectures, RNNs can also be trained using the BP method. A variant of the BP method called back propagation through time (BPTT) is the standard optimization method for RNNs [25], [103], and some alternative methods have also been proposed to speed up the optimization or to extend its capacity [63], [104], [105], [106], [107].

## Applications in biomedicine

Owing to advances in high-throughput technologies, a deluge of biological and medical data has been obtained in recent decades, including data related to medical images, biological sequences, and protein structures. Some successful applications of deep learning in biomedical fields are reviewed in this section and a summary of applications is shown in Table 1.

**Table 1:** **Applications of deep learning frameworks in biomedical informatics**

**Topic**	**DL architecture**	**Brief description**	**Refs.**
Medical images analysis	CNN	Brain tumor segmentation, won top 2 in BRATS	[108]
		Segmentation of pancreas in CT	[113]
		Knee cartilage segmentation	[114]
		Segmentation of hippocampus	[117]
		Predict semantic descriptions from medical images	[118]
		Segmentation of MR brain images	[121]
		Anatomy-specific classification of medical images	[123]
		Cerebral microbleeds from MR images	[125]
		Coronary artery calcium scoring in CT images	[126]
		Nuclei detection in routine colon cancer histology images	[129]
		Histopathological cancer classification	[130]
		Invasive ductal carcinoma segmentation in WSI	[132]
		Mammographic lesions detection	[133]
		Haemorrhages detection in fundus images	[137]
		Exudates detection in fundus images	[138]

	SAE	Segmentation of hippocampus from infant brains	[116]
		Organ detection in 4D patient data	[122]
		Histological characterization healthy skin and healing wounds	[124]
		Scoring of percentage mammographic density and mammographic texture related to breast cancer risk	[134]
		Optic disc detection from fundus photograph	[135]

	DBN	Segmentation of left ventricle of the heart from MR data	[112]
		Discriminate retinal-based diseases	[139]

	DNN	Brain tumor segmentation in MR images, won 2nd place in BRATS	[109]
		Prostate MR segmentation	[115]
		Gland instance segmentation	[119]
		Semantic segmentation of tissues in CT images	[120]
		Mitosis detection in breast cancer histological images	[131]

	RNN	EEG-based prediction of epileptic seizures propagation using time-delayed NN	[141]
		Classification of patterns of EEG synchronization for seizure prediction	[142]
		EEG-based lapse detection	[143]
		Prediction of epileptic seizures	[144]

Genomic sequencing and gene expression analysis	DNN	Gene expression inference	[145]
		Identification of *cis*-regulatory regions and replication timing domains	[151]
		Prediction of enhancer	[152]
		Prediction of splicing patterns in individual tissues and differences in splicing patterns across tissues	[159]
		Annotation of the pathogenicity of genetic variants	[161]

	DBN	Modeling structural binding preferences and predicting binding sites of RNA-binding proteins	[146]
		Prediction of splice junction at DNA level	[156]
		Prediction of transcription factor binding sites	[148], [149]
		Annotation and interpretation of the noncoding genome	[151]
		Prediction of the noncoding variant effects *de novo* from sequence	[162]

	RNN	Prediction of miRNA precursor and miRNA targets	[153], [154]
		Detection of splice junctions from DNA sequences	[157]
		Prediction of non-coding function *de novo* from sequence	[163]
		Analysis of human splicing codes and their determination of diseases	[161]

Protein structure prediction	DBN	Modeling structural binding preferences and predicting binding sites of RBPs	[146]
		*Ab initio* prediction of the protein secondary structures	[171]
		Prediction of protein disorder	[184]
		Prediction of secondary structures, local backbone angles, and solvent accessible surface area of proteins	[170]

	CNN	Prediction of protein order/disorder regions	[183]
		Prediction of protein secondary structures	[179], [180], [182]
		Prediction of protein structure properties, including secondary structure, solvent accessibility, and disorder regions	[185]

	SAE	Sequence-based prediction of backbone Cα angles and dihedrals	[169]

	RNN	Prediction of protein secondary structure	[172], [173], [174], [178]
		Prediction of protein contact map	[175], [176], [177]

*Note*: NN, neural networks; CNN, convolutional NN; SAE, stacked auto-encoder; DBN, deep belief network; RNN, recurrent NN.

### Medical image classification and segmentation

Machine learning for medical images has long been a powerful tool in the diagnosis or assessment of diseases. Traditionally, discriminative features referring to medical image interpretation are manually designed for classification (detection of lesions or abnormalities) and segmentation of regions of interest (tissues and organs) in different medical applications. This requires the participation of physicians with expertise. Nonetheless, the complexity and ambiguity of medical images, limited knowledge for medical image interpretation, and the requirement of large amounts of annotated data have hindered the wide use of machine learning in the medical image domain. Notably, deep learning methods have attained success in a variety of computer vision tasks such as object recognition, localization, and segmentation in natural images. These have soon brought about an active field of machine learning in medical image analysis.

Segmentation of tissues and organs is crucial for qualitative and quantitative assessment of medical images. Pereira et al. used data augmentation, small convolutional kernels, and a pre-processing stage to achieve accurate brain tumor segmentation [108]. Their CNN-based segmentation method won first place in the Brain Tumor Segmentation (BRATS) Challenge in 2013, and second place in 2015. Havaei et al. presented a fully automatic brain tumor segmentation method based on DNNs in magnetic resonance (MR) images with a two-phase training procedure [109], which obtained second place in the 2013 BRATS. Their methodology was tested on the publicly available datasets INbreast [110] and Digital Database for Screening Mammography (DDSM) [111], outperforming in terms of accuracy and efficiency several state-of-the-art methods when tested on DDSM. Additional medical applications employing a deep learning architecture have been demonstrated in segmenting the left ventricle of the heart from the MR data [112], the pancreas through computed tomography (CT) [113], tibial cartilage through magnetic resonance imaging (MRI) [114], the prostate through MRI [115], and the hippocampus through MR brain images [116], [117]. The differentiation of tissues or organs in medical images has been termed semantic segmentation [118], [119] in which each pixel of an image is assigned to a class or a label. The skeletal muscles, organs, and fat in CT images are well delineated through semantic segmentation based on a DNN architecture [120]. Similarly, the semantic segmentation of MR images also attained accurate segmentation results [121], [122], [123].

Detection of lesion and abnormality is the major issue in medical image analysis. Deep learning methods learn the representations directly instead of using hand-crafted features from training data. A classifier is then used to assign the representations to a probability that indicates whether or not the image contains lesions. In other words, the deep learning schemas classify each pixel to be a lesion point or not, which can be done in two ways: (1) classifying the mini patch around the pixel with a deep network, and (2) using a fully convolutional network to classify each pixel.

Sheet et al. [124] applied a DNN to histologically characterize healthy skin and healing wounds to reduce clinical reporting variability. Two unsupervised pre-trained layers of denoising AEs (DAEs) were used to learn features in their hybrid architecture, and subsequently the whole network was learned using labelled tissues for characterization. Detection of cerebral microbleeds [125] and coronary artery calcification [126] also produced better results when using deep learning-based approaches. In addition, brain tumor progression prediction implemented with a deep learning architecture [127] has also shown a more robust tumor progression model in comparison with a high-precision manifold learning approach [128].

Detection of pathologies on stained histopathology images [129], [130], [131] exemplify the high precision of deep learning-based approaches. For breast cancer detection in histopathology images, Cruz-Roa et al. [132] established a deep learning model to precisely delineate the invasive ductal carcinoma (IDC) regions to distinguish the invasive tumor tissue and non-invasive or healthy tissue. Their 3-layer CNN architecture, composed of two cascading convolutional and pooling layers, a full-connected layer, and a logistic regression classifier for prediction, attained a better F-measure (71.8%) and higher balanced accuracy (BAC; 84.23%) in comparison with an approach using handcrafted image features and a machine learning classifier.

The mammogram is one of the most effective imaging modalities in early diagnosis and risk prediction of breast cancer. A deep learning model [133] trained on a large dataset of 45,000 images attained performance similar to that of certified screening radiologists in mammographic lesion detection. Kallenberg et al. [134] investigated the scoring of percentage mammographic density (PMD) and mammographic texture (MT) related to prediction of breast cancer risk. They employed a sparse AE to learn deep hierarchical features from unlabeled mammograms. Multinomial logistic regression or softmax regression was then used as a classifier in the supervised training. As a result, the performance of their approach was comparable with that of the subjective and expensive manual PMD and MT scorings.

Color fundus photography is an important diagnostic tool for ophthalmic diseases. Deep learning-based methods with fundus images have recently gained considerable interest as a key to developing automated diagnosis systems. A DNN architecture was proposed by Srivastava et al. [135] to distinguish optic disc (OD) from parapapillary atrophy (PPA). A DNN consisting of SAEs followed by a refined active shape model attained accurate OD segmentation. For image registration, deep learning in combination with a multi-scale Hessian matrix [136] was used to detect vessel landmarks in the retinal image, whereas convolutional neural networks have also produced excellent results in the detection of hemorrhages [137] and exudates [138] in color fundus images. It is difficult to design an automatic screening system for retinal-based diseases such as age-related molecular degeneration, diabetic retinopathy, retinoblastoma, retinal detachment, and retinitis pigmentosa, because these diseases share similar characteristics. Through deep learning methods, Arunkumar et al. [139] successfully built a system to discriminate retina-based diseases only using fundus images. First, a DBN composed of a stack of RBMs was designed for feature extraction. Then a generalized regression neural network (GRNN) was employed to reduce dimensionality. Finally, a multi-class SVM was used for classification. Interestingly, Kaggle organized a competition on the staging of diabetic retinopathy from 35,126 training and 53,576 test color fundus images in 2015. Using convolutional neural networks, the top model outperformed other machine learning methods with a kappa score of 0.8496 (https://www.kaggle.com/c/diabetic-retinopathy-detection/leaderboard).

In addition to static images, time-series medical records such as signal maps from electro-encephalography and magnetoencephalography can also be analyzed using deep learning methods [140], [141]. These deep learning schemas take coded features of signals [142], [143] or raw signals [144] as input, and extract features from the data for anomaly classification or understanding emotions.

All the aforementioned applications illustrate that as a frontier of machine learning, deep learning has made substantial progress in medical image segmentation and classification. We expect that more clinical trials and systematic medical image analytic applications will emerge to help achieve better performance when applying deep learning in medicine.

### Genomic sequencing and gene expression analysis

Deep learning also plays an important role in genomic sequencing and gene expression analyses. To infer the expression profiles of target genes based on approximately 1000 landmark genes from the NIH Integrated Network-based Cellular Signatures (LINCS) program, Chen et al. presented D-GEX, a deep learning method with dropout as regularization, which significantly outperformed linear regression (LR) in terms of prediction accuracy on both microarray and RNA-seq data [145]. By applying a multimodal DBN to model structural binding preferences and to predict binding sites of RNA-binding proteins (RBPs) using the primary sequence as well as the secondary and tertiary structural profiles, Zhang et al. achieved an AUC of 0.98 for some proteins [146]. To predict binding sites of DNA- and RNA-binding proteins, Alipanahi et al. developed DeepBind, a CNN-based method, which surpassed other state-of-the-art methods, even when trained with *in vitro* data and tested with *in vivo* data [147]. Subsequently, Lanchantin et al. [148] and Zeng et al. [149] also applied CNN to predict transcription factor binding sites (TFBSs), and both studies demonstrated an improvement over the performance of DeepBind (AUC of 0.894). The input of these deep CNNs is encoded sequence characters obtained through protein binding microarrays or other assays, and the output is a real value indicating whether the sequence is a binding site or not. The deeper model can make more accurate classification by extracting higher-level features from the raw nucleotide sequences [148]. In addition, Kelley et al. presented Basset, an open source package to apply deep CNNs to learn the chromatin accessibility code, enabling annotation and interpretation of the noncoding genome [150]. Other applications include that of Li et al. [134] and Liu et al. [151], [152], who proposed deep learning approaches for the identification of *cis*-regulatory regions and replication timing domains, respectively. In addition, Yoon and his collaborators employed RNNs to predict miRNA precursors and targets. As a result, they achieved 25% increase in F-measure compared to existing alternative methods [153], [154].

Genetic variation can influence the transcription of DNA and the translation of mRNA [155]. Understanding the effects of sequence variants on pre-mRNA splicing facilitates not only whole genome annotation but also an understanding of genome function. To predict splice junction at the DNA level, Yoon and his collaborators developed a novel DBN-based method that was trained on the RBMs by boosting contrastive divergence with categorical gradients [156]. Their method not only achieved better accuracy and robustness but also discovered subtle non-canonical splicing patterns [156]. Furthermore, by exploiting RNNs to model and detect splice junctions from DNA sequences, the same authors also achieved a better performance than the previous DBN-based method [157].

Frey et al. formulated the assembly of a splicing code as a statistical inference problem [158], and proposed a Bayesian method to predict tissue-regulated splicing using RNA sequences and cellular context. Subsequently, they developed a DNN model with dropout to learn and predict alternative splicing (AS) [159]. This model took both the genomic features and tissue context as inputs, and predicted splicing patterns in individual tissues and differences in splicing patterns across tissues. They showed that their method surpassed the previous Bayesian methods and other common machine learning algorithms, such as multinomial logistic regression (MLR) and SVMs, in terms of AS prediction. Furthermore, they built a computational model using a Bayesian deep learning algorithm to predict the effects of genetic variants on AS [160]. This model took DNA sequences alone as input without using disease annotations or population data, and then scored the effects that variants had on AS, providing valuable insights into the genetic determinants of spinal muscular atrophy, nonpolyposis colorectal cancer, and autism spectrum disorder.

To annotate the pathogenicity of genetic variants, Quang et al. developed a DNN algorithm named DANN, which outperforms logistic regression (LR) and SVMs, with the AUC metric increased by 14% over SVMs [161]. Zhou et al. proposed a CNN-based algorithmic framework, DeepSEA, to predict the functional effects of noncoding variants *de novo* from sequences [162]. DeepSEA directly learns a regulatory sequence code from large-scale chromatin-profiling data, and can then predict the chromatin effects of sequence alterations with single-nucleotide sensitivity, and further prioritize functional variants based on the predicted chromatin effect signals. Subsequently, DanQ, a novel hybrid framework that combines CNN and bi-directional long short-term memory (BLSTM) RNNs, was presented to predict non-coding function *de novo* from sequences alone [163]. DanQ achieved an AUC 50% higher than other models, including the aforementioned DeepSEA.

### Prediction of protein structure

The 3D structure of proteins is determined by their comprising amino acid sequence [164]. However, the computational prediction of 3D protein structure from the 1D sequences remains challenging [165]. The correct 3D structure of a protein is crucial to its function, and improper structures could lead to a wide range of diseases [166], [167], [168]. Deep learning technologies have shown great capabilities in the area of protein structure prediction, which aims to predict the secondary structure or contact map of a protein.

Lyons et al. reported the first SAE for sequence-based prediction of backbone Cα angles and dihedrals [169]. Heffernan et al. also employed SAEs to predict secondary structure, local backbone angles, and solvent-accessible surface area (ASA) of proteins from amino acid sequences [170]; they achieved an accuracy of 82% for secondary structure prediction. Spencer et al. proposed DNSS, an *ab initio* approach to predicting the secondary structure of proteins using deep learning network architectures [171]. DNSS was trained using a position-specific scoring matrix of the protein sequence and Atchley’s factors of residues, and was optimized to accelerate the computation using the GPU and compute unified device architecture (CUDA). Baldi and his colleagues successfully applied various RNN-based algorithms to predict protein secondary structure [172], [173], [174] and protein contact map [175], [176], [177], with accuracies of 84% and 30%, respectively. Sønderby et al. used a bidirectional RNN (BRNN) with long short-term memory cells to improve the prediction of secondary structure, with better accuracy (0.671) than that using state of the art (0.664) [178]. Compared with SAEs, DBNs, and RNNs, CNNs were seldom used for protein structure prediction until recently. Li et al. developed Malphite, a CNN and ensemble learning-based method for predicting protein secondary structures, which achieved an accuracy of 82.6% for a dataset containing 3000 proteins [179]. Additionally, Lin et al. proposed MUST-CNN, a multilayer shift-and-stitch convolutional neural network architecture to predict protein secondary structure from primary amino acid sequences [180]. Besides classical deep learning architectures, some other architectures were also employed to predict protein secondary structure. For example, Lena et al. introduced a deep spatio-temporal learning architecture, achieved an accuracy roughly 10% higher than other methods [181], and Zhou et al. presented a deep supervised and convolutional generative stochastic network, achieving an accuracy of 66.4% [182].

In addition to the secondary structure prediction, deep learning was also employed in protein region prediction [183], [184]. For instance, sequenced-based predictor of protein disorder using boosted ensembles of deep networks (DNdisorder), a deep neural network with multi-layers of RBMs [184], achieved an average balanced accuracy of 0.82 and an AUC of 0.90. Incorporated with predicted secondary structure and predicted ASA, a weighted deep convolutional neural fields (DeepCNF) was proposed to predict protein order/disorder regions, obtains an AUC of 0.898 on the Critical As-sessment of Techniques for Protein Structure Prediction (CASP10) dataset [183]. All of these methods surpassed other state-of-the-art predictors in accuracy while still maintaining an extremely high computing speed. Recently, RaptorX-Property, a web server employing DeepCNF, was also presented to predict protein structure properties, including secondary structure, solvent accessibility, and disorder regions [185]. RaptorX-Property can be easily used and offer good performance (an AUC of 0.89 on its test data).

## Conclusion and perspective

**Figure 4:**
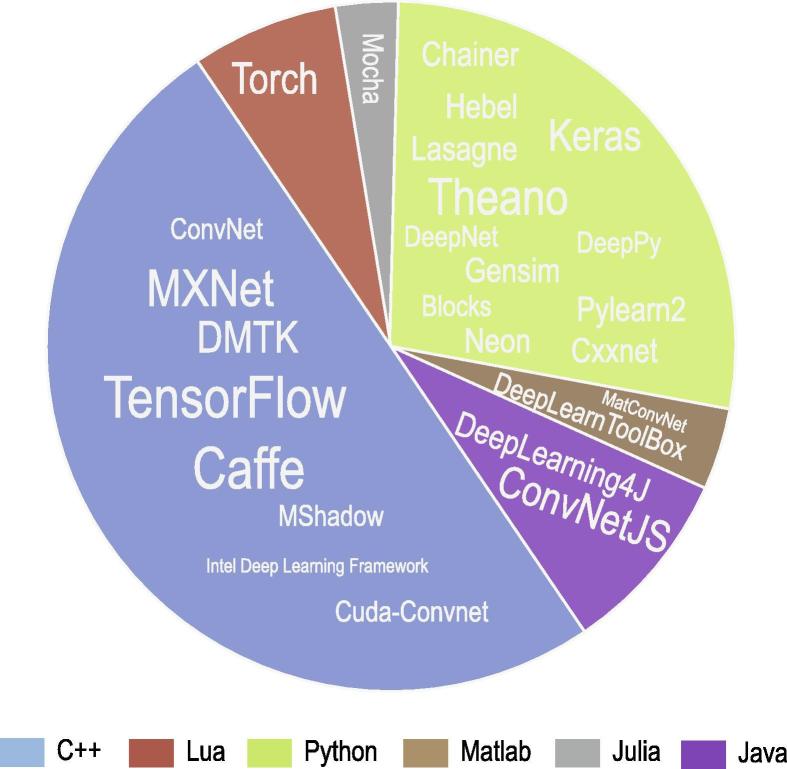
**Popularity of deep learning frameworks in Github** The distributions of stars in Github of deep learning frameworks written in C++, Lua, Python, Matlab, Julia, and Java are shown in the pie chart. More stars in Github indicate higher popularity. Font size of the frameworks in the pie chart reflects the number of stars.

Deep learning is moving toward its original goal: artificial intelligence. The state-of-the-art feature extraction capacity of deep learning enables its application in a wide range of fields. Many deep learning frameworks are open source, including commonly-used frameworks like Torch, Caffe, Theano, MXNet, DMTK, and TensorFlow. Some of them are designed as high-level wrappers for easy use, such as Keras, Lasagne, and Blocks. The applications of deep learning algorithms is further facilitated by the freely available sources. Figure 4 summarizes commonly-used frameworks in Github (https://github.com/) where the number of stars reflects the popularity of the frameworks.

Breakthroughs in technologies, particularly next-generation sequencing, are producing a large quantity of genomic data. Efficient interpretation of these data has been attracting much attention in recent years. In this scenario, uncovering the relationship between genomic variants and diseases, and illustrating the regulatory process of genes in cells have been important research areas. In this review, we introduced the way deep learning gets involved in these areas using examples. With deep architecture, these models can simulate more complex transformations and discover hierarchical data representations. On the other hand, almost all of these models can be trained in parallel on GPUs for fast processing. Furthermore, deep learning can extract data-driven features and deal with high-dimensional data, while machine learning usually depends on hand-crafted features and is suitable only to low-dimensional data. Thus, deep learning is becoming more and more popular in genomic sequence analysis.

Deep learning is represented by a group of technologies (introduced in brief description of deep learning), and has been widely used in biomedical data (introduced in applications in biomedicine). SAEs and RBMs can extract patterns from unlabeled data [186] as well as labeled data when stacked with a classifier [156]. They can also deal with dynamic data [187]. CNNs are most commonly used in the biomedical image analysis domain due to their outstanding capacity in analyzing spatial information. Although relatively few CNNs are used in sequencing data, CNNs have great potential in omics analysis [147] and biomedical signals [142]. On the other hand, RNN-based architectures are tailored for sequential data, and are most often used for sequencing data [154], [157] and in dynamic biomedical signals [144], but less frequently in static biomedical images. Currently, more and more attention is being paid to the usage of deep learning in biomedical information, and new applications of each schema may be discovered in the near future.

Despite the notable advantages of deep learning, challenges in applying deep learning to the biomedical domain still remain. Take biomedical image analysis for instance: we use fundus images to exemplify how deep learning works to define the level of diabetic retinopathy, and to detect lesion areas in different ways. Besides high accuracy and speed, the intelligent use of receptive fields also endows deep learning with overwhelming superiority in terms of image recognition. Furthermore, the development of end-to-end classification methods based on deep learning sheds new light on classifying pixels as lesioned or not. However, the usage of deep learning in medical images is still challenging. For model training, we need large amounts of data with labels, sometimes with labels in terms of pixel classification. Manually labeling these medical images is laborious and requires professional experts. On the other hand, medical images are highly associated with privacy, so collecting and protecting the data is demanding. Furthermore, biomedical data are usually imbalanced because the quantity of data from normal classes is much larger than that from other classes.

In addition to the balancing challenges, the large amount of data required, and the labeling for biomedical data, deep learning also requires technological improvements. Unlike other images, subtle changes in medical images may indicate disease. Therefore, analyzing these images requires high-resolution inputs, high training speed, and a large memory. Additionally, it is difficult to find a uniform assessment metric for biomedical data classification or prediction. Unlike other projects, we can tolerate false positives to some extent, and reject few or no false negatives in disease diagnosis. With different data, it is necessary to assess the model carefully and to tune the model according to characteristics of the data. Fortunately, the deeper networks with inception modules are accelerated [188], [189] and provide higher accuracy in biomedical image analysis [190]. On the other hand, crowdsourcing approaches have begun to pave the way in collecting annotations [191], [192], which may be an important tool in the next few years. These bidirectional drivers would promote the applications of deep learning in biomedical informatics.

As a long-term goal, precision medicine research demands active learning from all biological, biomedical, as well as health data. Together with medical devices and instruments, wearable sensors and smart phones are providing unprecedented amounts of health data. Deep learning is a promising interpreter of these data, serving in disease prediction, prevention, diagnosis, prognosis, and therapy. We expect that more deep learning applications will be available in epidemic prediction, disease prevention, and clinical decision-making.

## Competing interests

The authors have declared no competing interests.
